# CamurWeb: a classification software and a large knowledge base for gene expression data of cancer

**DOI:** 10.1186/s12859-018-2299-7

**Published:** 2018-10-15

**Authors:** Emanuel Weitschek, Silvia Di Lauro, Eleonora Cappelli, Paola Bertolazzi, Giovanni Felici

**Affiliations:** 1 Department of Engineering, Uninettuno International University, Corso Vittorio Emanuele II 39, Rome, 00186 Italy; 2 Institute of Systems Analysis and Computer Science “A. Ruberti”, National Research Council, Via dei Taurini 19, Rome, 00185 Italy; 3 Department of Engineering, Roma Tre University, Via della Vasca Navale 79, Rome, 00146 Italy; 4 SYSBIO.IT Center for Systems Biology, Milano Bicocca University, Piazza della Scienza 2, Milan, 20126 Italy

**Keywords:** Classification, Knowledge extraction, Big data, Cancer

## Abstract

**Background:**

The high growth of Next Generation Sequencing data currently demands new knowledge extraction methods. In particular, the RNA sequencing gene expression experimental technique stands out for case-control studies on cancer, which can be addressed with supervised machine learning techniques able to extract human interpretable models composed of genes, and their relation to the investigated disease. State of the art rule-based classifiers are designed to extract a single classification model, possibly composed of few relevant genes. Conversely, we aim to create a large knowledge base composed of many rule-based models, and thus determine which genes could be potentially involved in the analyzed tumor. This comprehensive and open access knowledge base is required to disseminate novel insights about cancer.

**Results:**

We propose CamurWeb, a new method and web-based software that is able to extract multiple and equivalent classification models in form of logic formulas (“if then” rules) and to create a knowledge base of these rules that can be queried and analyzed. The method is based on an iterative classification procedure and an adaptive feature elimination technique that enables the computation of many rule-based models related to the cancer under study. Additionally, CamurWeb includes a user friendly interface for running the software, querying the results, and managing the performed experiments. The user can create her profile, upload her gene expression data, run the classification analyses, and interpret the results with predefined queries. In order to validate the software we apply it to all public available RNA sequencing datasets from The Cancer Genome Atlas database obtaining a large open access knowledge base about cancer. CamurWeb is available at http://bioinformatics.iasi.cnr.it/camurweb.

**Conclusions:**

The experiments prove the validity of CamurWeb, obtaining many classification models and thus several genes that are associated to 21 different cancer types. Finally, the comprehensive knowledge base about cancer and the software tool are released online; interested researchers have free access to them for further studies and to design biological experiments in cancer research.

## Background

High throughput sequencing technologies represent a true revolution in the field of molecular biology. Particularly in cancer applications, Next Generation Sequencing (NGS) methodologies have given great impetus to biomedical research approaches in the perspective of personalized medicine [1–6]. Thanks to NGS based experiments, which are becoming cheaper and cheaper, a huge amount of biological data is being generated. However, these data must be collected, organized, and interpreted in order to be made available to the scientific community [7].

In this work, we focus on the RNA sequencing (RNA-seq) NSG experiment [8] for quantifying the gene expression across the transcriptome in a given tissue [9, 10]. Indeed, studying the quantification of the transcriptome enables to understand which genes are activated at different phases of the cell cycle or in the development of pathological conditions. We consider RNA-seq NGS experiments related to tumoral samples extracted from the Genomic Data Commons (GDC) [11], a web portal dedicated to cancer care and prevention, which is an evolution of the The Cancer Genome Atlas (TCGA) [12]. The GDC is the result of an initiative funded by the National Cancer Institute (NCI) [13] with the aim of creating a unified data system that can promote the sharing of genomic and clinical data among researchers. The GDC portal publicly provides dataset of the following genomic experiments of more than 40 tumor types: DNA sequencing, Copy Number Variation, Somatic Mutations, DNA Methylation Gene Expression Quantification, and miRNA Expression Quantification. These datasets are retrievable with: (i) The GDC Data Portal [14], a web portal that allows browsing, retrieving, and downloading genomic and clinical data; (ii) The GDC Data Transfer Tool [15], a standard client-based software for high performance batch access; (iii) The GDC Application Programming Interface (API) [16] that allows programming or command line access, for searching and downloading subsets of data files based on specific parameters. Thanks to these precious retrieval tools, the GDC provides researchers and medical doctors with the largest repository of tumoral data collected from thousands of patients, potentially allowing several analyses on all actually known tumor types. However, in order to fully exploit this big data repository, new methods for extracting knowledge are required [7].

Data mining, a set of techniques and methodologies for extracting knowledge from large amounts of data, is a natural way to approach this task [17, 18]. Data mining techniques and algorithms point to the identification of *patterns*, which can be repeated under certain conditions. For example, a genomic pattern, which can be crucial for verifying or even recognizing a pathological condition related to a particular disease, could be identified. When a certain relationship is identified in the data mining process, the concept of learning this relationship is considered and this process is called *machine learning* [19]. Learning can take place with different approaches; in this work, we consider supervised learning techniques [20] (i.e., classification), which can be applied when in the analyzed dataset each element is associated to a finite set of properties (also called *class*). In particular, we focus on rule-based classifiers, where the pattern to be found is a set of conditions for which a certain class can be assigned to a sample. The rules are therefore logic formulas that bind a subset of features of the samples to their class label. Example of a logic formula or (“if then” rule) on gene expression data is the following “*if ENSG00000167676.3 < 16.15 OR ENSG00000166819.10 < 15.28 then the sample can be classified as tumoral*”. Several rule-based machine learning methods are available for the analysis of gene expression data, e.g., [21–24]. The reader may find a more detailed survey of these methods in [10].

Among them, we focus on a new supervised learning method that is able to extract more knowledge in terms of classification models than state of the art ones, called Classifier with Alternative and MUltiple Rule-based models (CAMUR) [25]. CAMUR is designed to find alternative and equivalent solutions for a classification problem building multiple rule-based classification models. Standard classifiers tend to extract few rules with a small set of features for discriminating the samples, and interesting features may remain hidden from the researcher. Thanks to an iterative classification procedure based on a feature elimination technique, CAMUR finds a large number of rules related to the classes present in the dataset under study. CAMUR is based on: (i) a rule-based classifier, i.e., RIPPER (Repeated Incremental Pruning to Produce Error Reduction) [26]; (ii) an iterative feature elimination technique; (iii) a repeated classification procedure; (iv) a storage structure for the classification rules. The method calculates iteratively a rule-based classification model through the RIPPER algorithm [26], deletes iteratively the features that are present in the rules from the dataset, and performs the classification procedure again, until a stopping criterion is met, i.e., the classification performance is below a given threshold or the maximum number of iterations set by the user has been reached. CAMUR has been implemented particularly for RNA-seq classification and case-control studies, i.e., specific studies that aim to identify subjects by their outcome status (e.g., tumoral or normal). In these data, the features correspond to the gene expressions of the samples, the classes to the investigated diseases or conditions (e.g., tumoral, normal). The extracted knowledge by CAMUR consists in a set of rules composed of a given number of genes that might be relevant for a disease. CAMUR also includes an offline tool to analyze and to interpret the computed results. Thus the software consists of two parts: (i) The Multiple Solutions Extractor (MSE), which corresponds to the implementation of the iterative classification algorithm (i.e., for each iteration it deletes the selected features, performs the classification, and saves the extracted models); (ii) The Multiple Solutions Analyzer (MSA), which is the graphical tool for analyzing and interpreting the obtained results. CAMUR is available at http://dmb.iasi.cnr.it/camur.php as stand alone software; for a comprehensive description we point the reader to [25].

In this work, we propose CamurWeb, a web implementation of CAMUR that is able to extract multiple rule-based classification models from RNA sequencing experiments and to create a large knowledge base of these rules. Moreover, we apply CamurWeb to all public RNA sequencing datasets extracted from The Cancer Genome Atlas database, obtaining a large open access knowledge base of classification rules related to several cancer types. Thanks to its user friendly interface, the tool allows to execute the software CAMUR, to query the results, and to manage the analyzed experiments.

## Implementation

This section introduces CamurWeb, the application designed and developed in this work. CamurWeb is a web service that aims to make the CAMUR software easily accessible and usable. CAMUR was developed in 2015 for the analysis and classification of genomic data, in particular to classify RNA-seq experiments and to extract an interesting body of rule-based classification models. The software and its algorithm are presented briefly at the end of the Background section. CAMUR has two main innovative aspects with respect to many machine learning algorithms: i) it derives many possible classification models and ii) it stores them to allow further and deeper analyses.

CamurWeb is designed to support these two aspects, making easy to exploit these two powerful functionalities even for a non specialized user. Before the release of CamurWeb, in order to run CAMUR the following tasks had to be performed by the user:

- install and configure a valid Java Virtual Machine [27];
- install and configure a MySQL database management system [28];
- download the CAMUR software package composed of the Multiple Solutions Extractor (MSE) and the Multiple Solutions Analyzer (MSA);
- start the MSE via the command line with its parameters;
- wait for the execution to complete;
- start MSA via the command line, and save the results of CAMUR by querying the interface.

These steps require time and effort and a good knowledge of computer usage. Conversely, CamurWeb allows using CAMUR in a fast and an intuitive way with a simple interface, directly through the browser without the need to install software or dealing with configurations. In the next paragraphs, we will describe the application requirements, and then deepen the architecture and its development.

### CamurWeb portal

The CamurWeb portal supports three main tasks:

- it permits to freely access, query, and visualize the large knowledge base of classification results (datasets, logic formulas, performance, and statistics) obtained running CAMUR on all public available RNA sequencing datasets of TCGA extracted from GDC;
- it enables the users to run the software online and to view the results of their classification analyses;
- it allows to download the CAMUR software package.

**Fig. 1:**
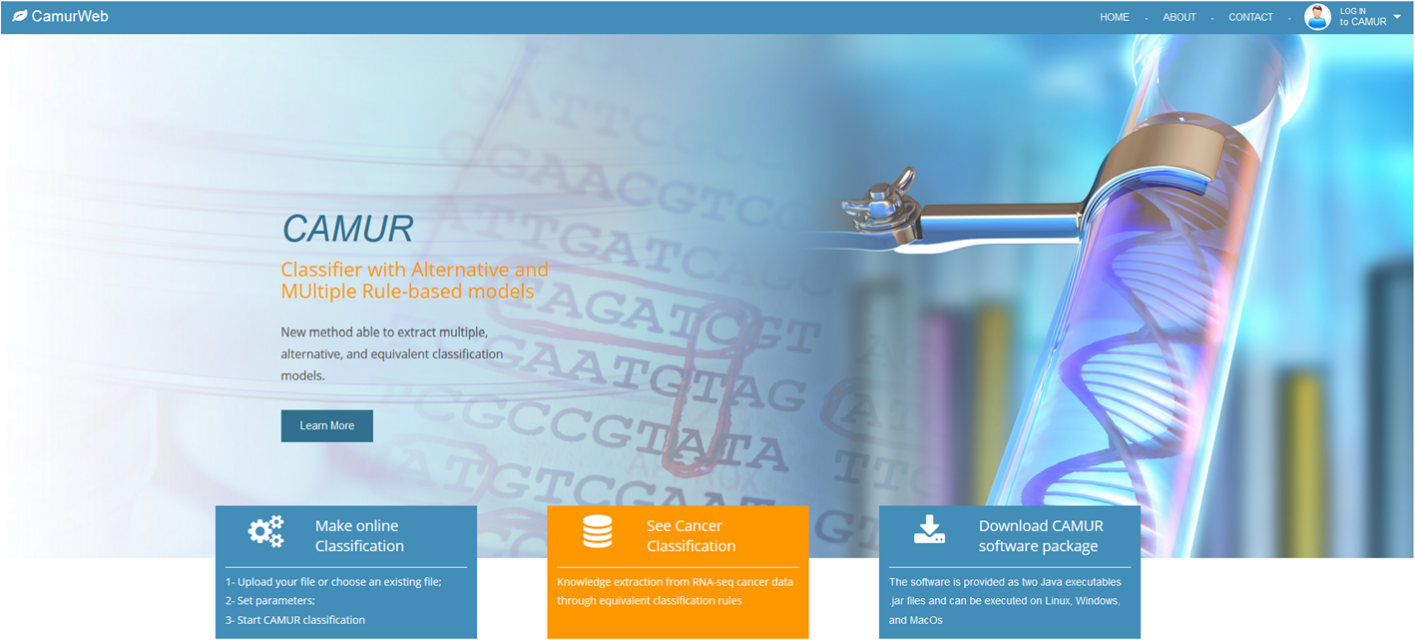
The homepage of CamurWeb

Therefore, CamurWeb home page is composed of three main sections, as depicted in Fig. 1: in the first one the users can perform the classification analyses, in the second one they can view the public analyses performed on the cancer datasets extracted from TCGA, and in the third one they can download the CAMUR software package.

The main users of CamurWeb can be of two types: the unregistered user, who can mainly access to the public results and repository about cancer; the registered one, who can run the classification software, save the performed analyses, and view her private as well as the public results.

In particular, the unregistered user can (i) learn and deepen the CAMUR classification tool: a section of the website is dedicated to briefly present the software and the web platform, and redirects the user to the bibliographic and web resources that deepen CAMUR; (ii) view the results of the classification analyses performed on 21 cancer datasets extracted from the Genomic Data Common (GDC) portal (detailed results of these analyses will be presented in “Results and discussion” section); (iii) ask for additional information or custom solutions through a simple form; (iv) sign up to the system simply by specifying an email and a password.

**Fig. 2:**
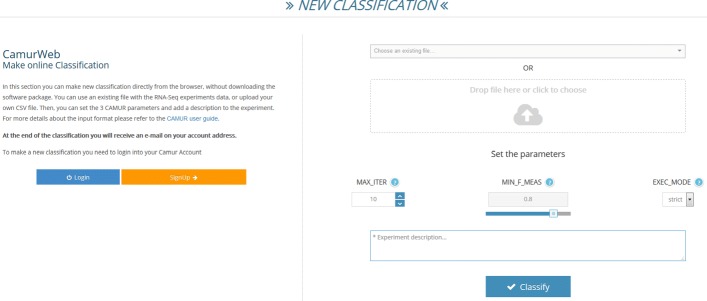
The classification section of CamurWeb

The registered user can perform all the previous operations and additionally has the possibility to: (i) perform a classification analysis with CAMUR by using a wizard, which allows to upload a dataset or choose from a set of existing ones containing data extracted from the GDC portal, set the parameters, and run the classification; (ii) view the classification results, i.e., the rule-based classification formulas, charts, and tables; then the user can query the database to see the results and export them; (iii) see a personal section with a report of the analyses started on the system and with her profile information. In order to run a classification task on a private dataset (see Fig. 2) the user must be registered. The system alerts the user with an e-mail at the end of the execution. This is another strength of CamurWeb, because processing a dataset with CAMUR can take few minutes to hours; so the user does not have to wait for the end of the execution connected to the system or with her computer turned on. The input file format of the CamurWeb classification online procedure is a standard comma separated values (csv) text file containing the data matrix of the RNA-seq experiments. For further details about the input format, we point the reader to the user guide of CAMUR available at http://dmb.iasi.cnr.it/camur.php.

**Fig. 3:**
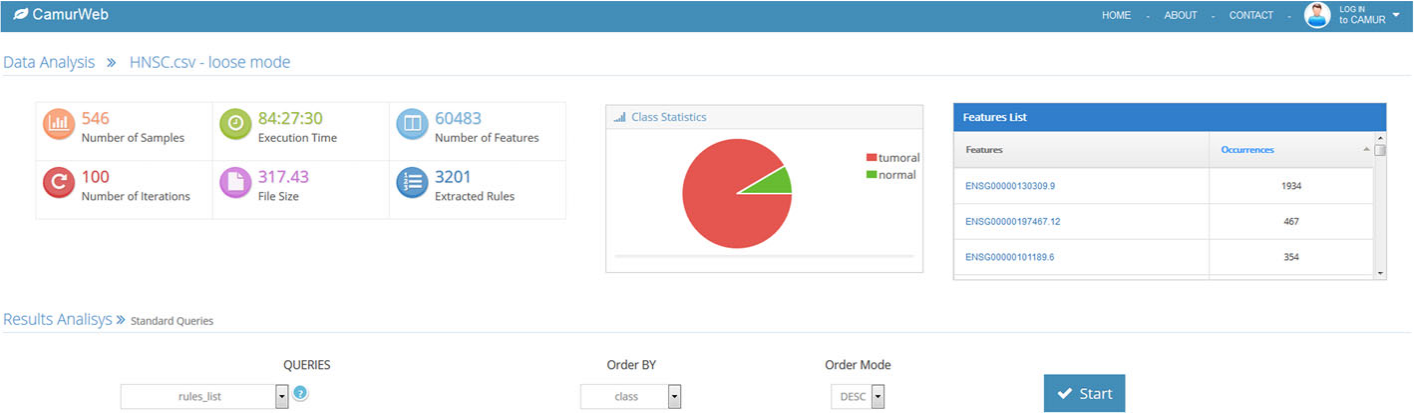
The results page of CamurWeb
- Literals and conjunctions list: identifies the conjunctions of the literals present in the rules; for each one the number of correct and incorrect instances and their percentages are returned;
- Rules list: extracts the literal disjunctions with their precision and accuracy;
- Literals statistics: returns more detailed statistics on the extracted genes and their thresholds;
- Feature pairs: extracts the pairs of genes present in the same rule and counts how many times they appear together.

The results of the access to the knowledge base, either the public or the private ones produced by running CAMUR, are reported on a *results page* (see Fig. 3). In this page CamurWeb shows: (i) a table with information about the uploaded file and the experiment, in particular the number of rows, which corresponds to the number of samples; the number of columns, which corresponds to the number of features; the size of the file; the time it took for the classification; the number of iterations chosen for the classification, and the number of extracted rules; (ii) a pie chart with the classes in the dataset with the percentage and number of samples; (iii) another table with the list of features extracted by the classifier and their number of occurrences; if the features are genes contained in the Ensembl database [29], the link leads to the page at www.ensembl.orgwith a description of the genes. Additionally, in the same page the user can perform the following knowledge extraction queries:

- Features List: extracts the list of genes and their occurrences in all the classification models obtained in the considered analysis;

The results of such queries can be visualized or downloaded.

### Tools and technologies

This section briefly presents the technologies and tools used for the CamurWeb application development.

CamurWeb is written in the Javascript programming language [30], which is suited not only for client-side applications but also for server-side ones. The Node.js framework [31] is adopted in this project. Node.js is a platform created on the Javascript engine, which allows to create fast and scalable web applications. The main features of Node.js are (i) an orientation towards the development of asynchronous code; (ii) a modular architecture; (iii) an optimized transmission of information through the HTTP connection. In addition to the APIs provided by Node.js, we use the Express.js library [32], a Node.js based framework that offers a robust set of functionalities to easily build single-page, multi-page, and hybrid web applications. It is a mature framework that offers several features including middleware, routing, the ability to manage application configurations in an easy way, and a template engine. Moreover, JQuery [33], a JavaScript library for web applications, is adopted in CamurWeb. It is born with the goal of simplifying selection, manipulation, event management, and animation in HTML pages. The jQuery library allows us to simplify JavaScript by writing complex instructions in one line. Additionally, the Bootstrap JavaScript library [34] is used for the development of the web interface. For managing the different executions of CAMUR, we adopt the REmote DIctionary Server (REDIs) [35], which is one of the most popular key-value databases. In CamurWeb, REDIs is used in Node.js for supporting the development of execution queues. It is used to handle a queue for CAMUR executions requested by the users. The maximum number of parallel executions of CAMUR is set in the application configuration file: a job being in the queue only starts if the number of active runs of CAMUR is less than the maximum number, otherwise the job is entered in the queue. Finally, CamurWeb uses MySql [28] as database management system in order to store the users identification data and the results of their analyses. In particular, the structure designed and used by CAMUR has been extended with new tables for the purposes. The MySql library is integrated in Node.js.

### Software architecture

CamurWeb follows the standard client-server model, i.e., the reference architecture for web applications [36]. In particular, CamurWeb uses is the *Model-View-Controller* (MVC) architectural pattern that allows to decouple the different components of the application to gain benefits in terms of reusability and maintenance [37]: *Model* contains data access methods; *View* takes care of displaying data to the user and manages the interaction between the user and the underlying infrastructure; *Controller* receives user commands across *View* and reacts by performing operations that may affect the *Model* and which generally lead to a View state change.

**Fig. 4:**
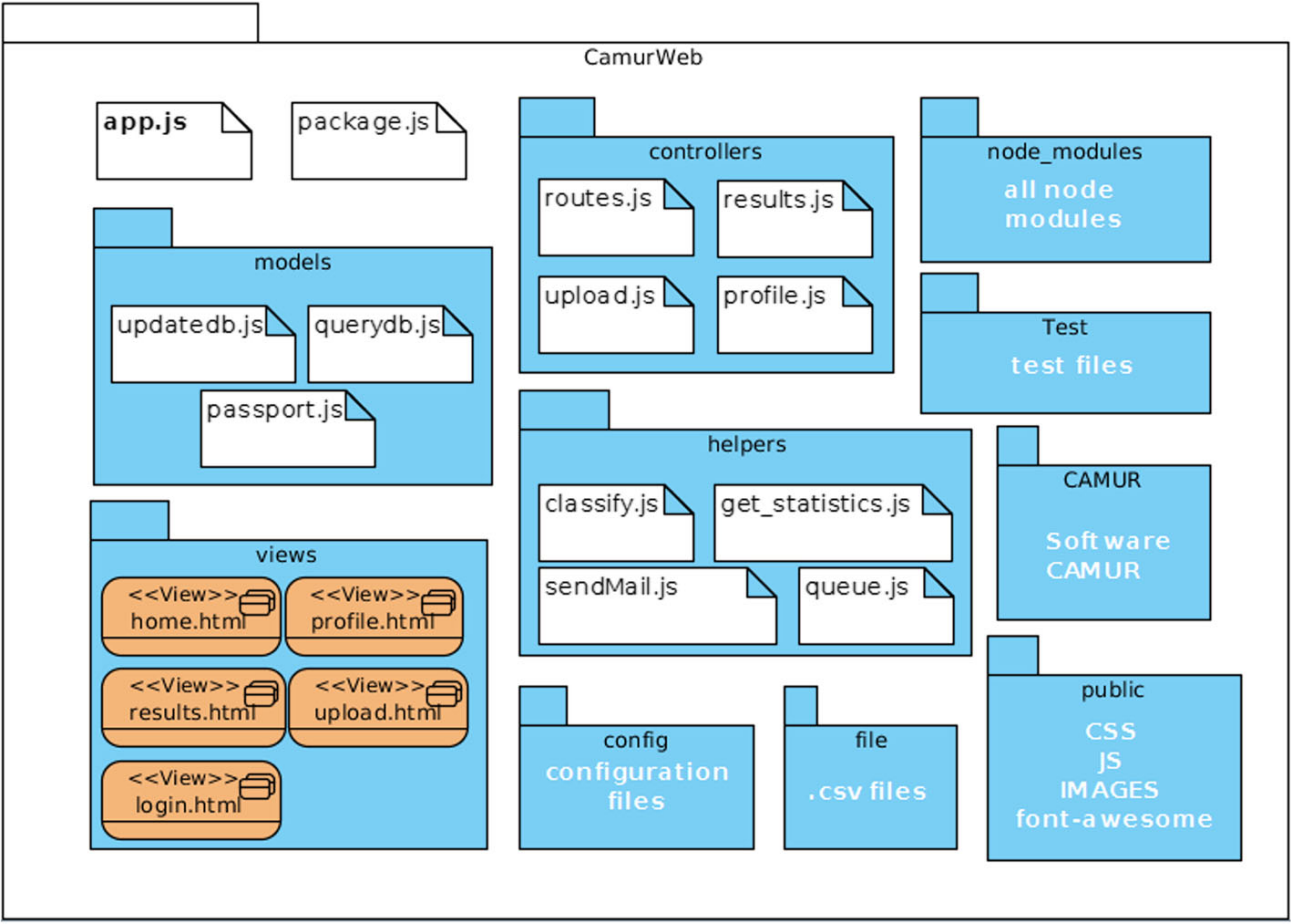
The software architecture of CamurWeb

The software architecture of CamurWeb is shown in Fig. 4 and described in the following. The software is composed of four main components and six other stand alone software modules. The *Controllers* component contains the routes of the application. Routes play a primary role: their job is to translate the different request urls by addressing the call to the correct function on the server. The *Views* component contains the software modules that constitute the web application interface described more in detail in “CamurWeb portal” subsection. The *Models* component contains the software modules that interact with the database. All operations that need to retrieve data from the database, insert, or update it, are handled by these modules. The *Helpers* component contains support software modules for the web application, e.g., the statistics functions, the send email facility, and the CAMUR executor. Finally, six additional stand alone modules are part of the software architecture: the node modules, which group the system libraries of Node.js; the config module, which contains the configuration files of the software; the CAMUR module, which contains the CAMUR software package; the public module, which contains useful files for the GUI; the file module, which manages the storage of the users’ file and of the public datasets; and lastly the test module, which manages the public analyses and the private ones performed by the different users.

## Results and discussion

In order to prove the validity of CamurWeb, we performed a classification analysis on all public available RNA sequencing datasets of The Cancer Genome Atlas database extracted from the Genomic Data Commons portal. For each dataset we obtain a large body of accurate classification models, which are composed of rule-based classification formulas containing many genes and their association to a particular cancer type. With these models we build a large knowledge base about cancer focusing on the extracted genes. Interested researchers and medical doctors can access these knowledge on our public section “See cancer classification” available at http://bioinformatics.iasi.cnr.it/camurweb. In the following, we describe the analyzed data and some of the obtained results.

### Analyzed data

The data selected for the analyses are extracted from the Genomic Data Commons (GDC) portal through its APIs [16] (scripts to download ad process data are available upon request). GDC collects, standardizes, and makes accessible large amounts of genomic and clinical data with the purpose of favoring and helping researchers to cure and prevent cancer. For more details about GDC, we point the reader to “Background” section and to [11]. In this study we focus on data of RNA-seq, which provides a comprehensive view of the transcripts of a cell, can identify new transcripts, is able to monitor splicing events, and permits to quantify gene expression. For this reason RNA-seq is considered a valid tool for a deep understanding of tumor processes. Therefore we select from the GDC portal all publicly available RNA-seq TCGA data, which are composed of gene expression measures on 9030 diseased and healthy tissues (92.6% and 7.4%, respectively). These data are obtained by adopting the Illumina HiSeq 2000 RNA Sequencing Version 2 (RNA-seq V2) platform [38] and are collected in GDC by the Cancer Genomic Characterization Center (CGCC) University of North Carolina. The public available tumors are 30, each one consisting of a set of samples taken from healthy tissues or diseased ones: healthy tissues are labeled in GDC with the term “normal” and diseased ones with the term “tumoral”. For each tissue GDC provides 60,483 gene expression values expressed with the *Fragments Per Kilobase per Million mapped* (FPKM) measure [39–41].

In order to be classified, the downloaded data are processed and transformed into a matrix format. We build a matrix for each tumor containing the FPKM gene expression values: the rows correspond to the samples, which range from 45 for the CHOL tumor to 1222 for BRCA; the first column represents the sample identifier; the central columns correspond to the 60,483 genes, whose expression is measured and which are identified by their Ensembl ID [29]; the last column represents the class of the sample (normal or tumoral); the element *c*_*ij*_ contains the FPKM value of the sample *i* measured on the gene *j*. An example of data matrix is shown in Table 1. Scripts for the conversion and assembly of the GDC data to a matrix format are available upon request. The input of CamurWeb is am RNA-seq matrix encoded in a comma separated values (csv) text file. In Table 2 we show the main characteristics of the obtained matrices. As the reader can see, RNA-seq experiments of cancers ACC, DLBC, LAML, LGG, MESO, OV, TGCT, UCS, and UVM only include samples of tumoral tissues. Therefore it is not possible to perform a supervised classification analysis of such cancer datasets.

**Table 1:** An example of RNA-seq data matrix

Aliquot	ENSG00000 130309.9	ENSG00000 101189.6	.........	ENSG00000 260597.1	Class
TCGA-4G..	0	9,7872338	....	0,141	Tumoral
TCGA-W5..	0,0323	1,4725	...	0,62107	Normal
......	.....	.....	.....	.....	....
TCGA-ZH..	0,06223	8,7757	.....	0,4818	Tumoral

Rows are indexed by the tissues, columns by the genes (except the last one containing the class). Each element of the matrix represents the FPKM gene expression value associated to the respective gene and tissue

**Table 2:** The considered data of The Cancer Genome Atlas extracted from the Genomic Data Commons portal

Cancer	# of tissues	# of tumoral	# of normal	% of tumoral	File size (MB)
ACC	79	79	0	100	45,08
BLCA	433	414	19	95,61	250,69
BRCA	1222	1102	120	90,18	592,77
CESC	309	304	5	98,38	180,67
CHOL	45	36	9	80,00	26,49
COAD	521	478	43	91,75	293,15
DLBC	48	48	0	100	28,62
ESCA	173	161	12	93,06	117,00
GBM	174	156	18	89,66	107,08
HNSC	546	500	46	91,58	317,43
KICH	89	65	24	73,03	52,83
KIRC	611	538	73	88,05	372,75
KIRP	321	288	33	89,72	187,99
LAML	173	173	0	100	98,28
LGG	534	534	0	100	319,55
LIHC	424	371	53	87,50	233,13
LUAD	594	533	61	89,73	353,07
LUSC	551	502	49	91,11	333,09
MESO	86	86	0	100	50,96
OV	309	309	0	100	238,69
PAAD	182	177	5	97,25	108,34
PCPG	186	178	8	95,70	107,82
READ	177	166	11	93,79	100,34
SARC	265	259	6	97,74	152,34
STAD	407	375	32	92,14	268,86
TGCT	156	156	0	100	95,25
THYM	121	119	2	98,35	72,01
UCEC	587	551	36	93,87	336,61
UCS	56	56	0	100	34,28
UVM	80	80	0	100	43,96

The number of tissues, the ratio of tumoral and normal ones, and the file size in MB is reported for each cancer dataset

### Classification analyses and creation of the knowledge base

We performed the classification analyses through the CamurWeb platform on all datasets containing normal and tumoral tissues. The parameters of CAMUR have been set as follows: the execution mode to *loose*, the maximum number of iterations to 100 and the minimum F-measure value to 0.8. The execution mode indicates how CAMUR runs, the loose mode is slower than the strict one, because computational complexity grows exponential to the number of features. On the other hand the loose mode permits to extract more knowledge with greater accuracy (F-measure). The maximum number of desired iterations of CAMUR is set to 100; this means that CAMUR is going to perform 100 runs each one with several classification procedures. The minimum F-measure is the value below which the classification results are not considered. CAMUR will stop after the maximum number of iterations has been reached or if the F-measure of all current runs is below the given threshold. For further details about the parameters setting, the reader may refer to [25]. The classification analyses have been performed on an Intel i7 workstation with 24 GB of RAM and by using the CentOs 7 64bit linux operating system with kernel 3.10.0-514.26.2.el7.x86_64. We executed 3 analyses concurrently. A total of 21 analysis tasks have been accomplished, resulting in more than 10,000 classification procedures.

Table 3 shows the results in terms of running time, number of inferred rules, and number of extracted genes (features). By comparing the results reported in Table 3 with the characteristics of the datasets shown in Table 2, we can draw some considerations regarding the link between the number of samples of the dataset and the execution time. The running time of CAMUR is not directly proportional to the number of samples (the number of rows) of the considered dataset. The number of samples actually affects only execution time of a single iteration of the CAMUR classifier; what determines the total time of the execution is the number of iterations. CAMUR continues its iterations since one of the stopping criteria is verified: (i) the maximum number of iterations imposed by the user is reached; (ii) the F-measure values are smaller than the threshold set by the user; (iii) all possible combinations are eliminated from the set of features.

**Table 3:** Results of the classification analyses with CamurWeb

Cancer	Execution time	# of iterations	# of rules	# of genes
BLCA	4:36:52	100	334	164
BRCA	190:29:57	30	3015	1847
CESC	0:01:50	20	5	3
CHOL	0:00:13	47	3	2
COAD	1:48:12	100	90	32
ESCA	0:56:09	100	229	122
GBM	14:21:12	100	1487	832
HNSC	84:27:30	100	3201	1363
KICH	0:00:52	26	8	5
KIRC	6:36:45	100	470	183
KIRP	0:01:17	9	3	2
LIHC	24:08:10	100	1890	854
LUAD	12:06:36	100	775	298
LUSC	0:06:23	32	8	5
PAAD	0:29:37	100	132	71
PCPG	6:35:40	100	348	173
READ	0:01:11	23	6	5
SARC	7:42:24	100	358	164
STAD	2:04:16	100	416	243
THYM	0:00:19	14	3	3
UCEC	3:52:26	100	496	209

We report for each considered cancer the execution time, the number of performed iterations, the number of extracted rules and genes by CAMUR

The fastest analyses, where not all 100 iterations are executed, are CESC, CHOL, KICH, KIRP, LUSC, READ, and THYM. In fact, in these analyses a small number of rules are extracted and consequently a small set of relevant genes is obtained. The cause can be a combination of the stopping criteria (ii) and (iii): it is possible that the rules extracted after the first iterations do not exceed the minimum value of F-Measure (0.8), and hence all their genes are not considered. The consequence is that the set of genes does not increase and the combinations to be eliminated from the original dataset quickly becomes empty.

It is worth to note that for the BLCA, BRCA, GBM, HNSC, KIRK, LIHC, LUAD, PCPG, SARC, STAD, UCEC tumors CAMUR extracted a high number of rules and many features (genes) that are potentially involved in the tumoral processes. For the other tumors CAMUR extracted a smaller set of genes that are related to the cancer under study.

**Fig. 5:**
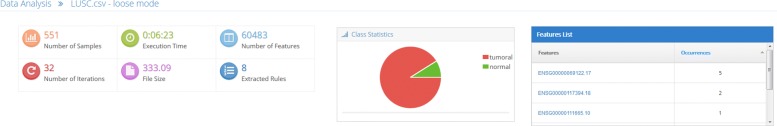
The results page of the classification analyses on the LUCS tumor

As an example Fig. 5 shows the results page of the classification analysis on the LUSC tumor. The reader can see that among the extracted features the ADGRF5 gene with Ensembl ID ENSG00000069122.17 is the one that occurs most in the classification rules. Previous studies have already shown that mutations within this gene are possible causes of lung cancer (LUSC) [42]. Similarly, many other genes extracted from the classification rules of LUSC are listed in several publications that concern this tumor [43].

The CHOL and KICH tumors are characterized by a small set of tissues (45 and 89) though with a percentage of normal ones greater than others. The classification analyses on these two tumors did not produce many rules, but for all the extracted ones the F-Measure and the accuracy was 1, i.e., no classification errors occurred.

Other examples and some considerations are reported in the following.

#### Head and Neck squamous cell carcinoma (HNSC)

HNSC is one of the analyses with higher execution time, because the CAMUR software was able to run 3201 classification procedures producing rules with accuracy values ranging from 0.95 to 1 and extracting 1363 genes. In Table 4 we report the genes that are most represented in the rules. We can see that the COLGALT1 gene with Ensembl ID ENSG00000130309.9 is the one that appears in the largest number of rules (1934 rules out of 3201). By examining more deeply the rules, this gene has an FPKM value above 18.16 in all tumoral tissues. Similar observations can be made for the genes COL13A1 (ENSG00000197467.12), MRGBP (ENSG00000101189.6), and following. Such examinations can be at a basis for targeted research and studies about cancer. Another investigation can be made with CamurWeb by studying pairs of genes that appear often together in the classification rules. This information can be obtained from the CamurWeb database with a simple query called “feature pairs”. We report part of the results for the HNSC tumor in Table 5. As the reader can see, the genes COLGALT1 (ENSG00000130309.9) and AC012531.25 (ENSG00000260597.1) is the most frequent couple that appears in the rules occurring 250 times. In particular, AC012531.25 is always extracted together with COLGALT1, because its number of occurrences as single gene is exactly 250. Even this investigation generates important results in helping to understand the genetics of cancer.

**Table 4:** Most represented genes in the rules extracted from the HNSC tumor

Gene	Occurrences
ENSG00000130309.9	1934
ENSG00000197467.12	467
ENSG00000101189.6	354
ENSG00000260597.1	250
ENSG00000197766.6	218
...	...

**Table 5:** Pairs of genes that occur most in the classification rules related to the HNSC tumor

Gene 1	Gene 2	Occurrences
ENSG00000260597.1	ENSG00000130309.9	250
ENSG00000130309.9	ENSG00000197766.6	203
ENSG00000256229.6	ENSG00000130309.9	167
ENSG00000164114.17	ENSG00000130309.9	165
...	...	...

#### Liver hepatocellular carcinoma (LIHC)

For this tumor CAMUR has identified 854 genes by running 1890 classification procedures. In this dataset the percentage of normal tissues (12.5%) is higher than in other tumors. In Table 6 we show the most represented genes that occur in the rules. It is worth noting that the GABRD (ENSG00000187730.7) gene is the most represented one, followed by the TOMM40L (ENSG00000158882.11) gene. Existing studies on the GABRD gene confirm that alterations in its expression can play a key role in differentiating tumor cells. In particular, an abnormal regulation leads to its overexpression that can cause the proliferation of tumor cells [44]. Regarding the second gene, a study has been published that relates the alteration of TOMM40L expression to the excess of smoke in humans [45]. In this study, the authors relate the effect of smoke and the elevated expression of TOMM40L by concentrating on neurodegenerative diseases such as Alzheimer’s and Parkinson’s. The findings of CamurWeb can be objective of future studies on this gene (and on other ones) that focus on cancer.

**Table 6:** Most represented genes in the rules extracted from the LIHC tumor

Gene	Occurrences
ENSG00000187730.7	413
ENSG00000158882.11	376
ENSG00000231856.2	295
ENSG00000164283.11	229
...	...

#### Breast Invasive Carcinoma (BRCA)

Analyses on the BRCA dataset are particularly interesting for the large number of available tissues (1222, 1102 tumoral, and 120 normal). Breast cancer is the most common tumor in the female population and represents 29% of all tumors affecting women. For this reason it is deeply studied, and we can find in literature a lot of findings about it. CAMUR executed 30 iterations on the BRCA dataset producing 3015 rules and extracting 1847 genes with a running time of 190 hours and 29 minutes. In Table 7 we report the most frequent genes that are present in the obtained classification rules. We highlight that previous research confirms the relationship between the alteration of the expression of the first three most occurring genes - SPRY2 (ENSG00000136158.9) [46], VEGFD (ENSG00000165197.4) [47], and MMP11 (ENSG00000099953.8) [48] - and the predisposition to Breast Cancer.

**Table 7:** Most frequent genes in the rules extracted from the BRCA tumor

Gene	Occurrences
ENSG00000136158.9	1078
ENSG00000165197.4	993
ENSG00000099953.8	725
ENSG00000157766.14	515
...	...

## Conclusions

In this work, we described CamurWeb, a new web portal for classifying NGS data of RNA sequencing and for sharing the obtained results. CamurWeb is a web application based on NodeJs, ExpressJs, and MySQL, which makes use of the CAMUR classification software. CAMUR is able to compute a large body of knowledge by finding a high number of genes that are likely to be involved in the processes that cause the formation of tumors. Conversely, state of the art rule-based classifiers extract from a dataset a set of two or three rules that describe it. However, this small set of rules may be insufficient to describe the data in a comprehensive way and to extract sufficient knowledge from it.

In order to prove the validity of CamurWeb and to release a large knowledge base of classification rules about cancer, we performed a wide supervised analysis on gene expression data belonging to more than 9000 patients and 21 different tumor types of The Cancer Genome Atlas extracted from the Genomic Data Commons portal. The obtained results were evaluated in terms of performance, execution times, and extracted features (genes related to a particular type of tumor). Among those genes, we identified a part of them already linked to the literature about cancer, confirming our classification procedure, and another part that still has to be investigated; this could be the starting point for new research studies. The identified genes can act as possible diagnostic and prognostic markers or therapeutic targets. All the extracted knowledge, the classification results, and the selected genes have been made public on the CamurWeb platform and can be consulted or queried for further investigation by biologists, medical doctors, and bioinformaticians in order to prove their association to a particular cancer.

Topics of future work may concern both the extension of the performed analyses and the development of new features for the CamurWeb application. Regarding the analyses, we plan to (i) investigate the role of the extracted genes for the different analyzed tumors and to compare them with existing studies; (ii) perform a set theoretic analysis of the extracted logic formulas in order to find common biomarkers among the studied cancers; (iii) repeat the classification analyses with the same data, but using different parameters, and then compare the results both in terms of extracted features, execution time, and accuracy of the rules; (vi) perform other classification analyses with new data extracted from other gene expression databases (e.g., GEO [49]) or projects (e.g., TARGET); (v) increase the number of public analyses, using other input or other classification parameters.

Regarding the CamurWeb platform we plan to: (i) design and develop automatic procedures able to integrate, compare, and analyze the logic classification formulas stored in the database; (ii) add a feature that allows users to share their own analyses; (iii) expand the user profile page by entering a field that allows the user to add observations or personal considerations about the analyses; (iv) increase the number of queries that can be made on the results database produced by CAMUR.

To conclude, we wish to highlight that the CamurWeb software and the published knowledge base are promising research tools for performing analyses on new released data and for discovering novel insights about cancer.

## Availability and requirements

**Project name:** CamurWeb. **Project home page:**http://bioinformatics.iasi.cnr.it/camurweb. **Operating system(s):** Windows, Linux, and MacOs. **Programming language:** Javascript and Java. **Other requirements:** An updated version (starting from 2016) of Firefox or Internet Explorer or Chrome. **License:** GNU General Public License, version 3 (GPL-3.0). **Any restrictions to use by non-academics:** Licence needed.

## Abbreviations

API: Application program interface
BED: Browser extensible data
BRCA: Breast invasive carcinoma
CNV: Copy number variation
COAD: Colon adenocarcinoma
CSV: Comma separated values
DLBC: Lymphoid neoplasm diffuse large B-cell lymphoma
DNA: Deoxyribonucleic acid
ESCA: Esophageal carcinoma
FTP: File transfer protocol
GBM: Glioblastoma multiforme
GDC: Genomic data commons
GFF: General feature format
GMQL: GenoMetric query language
GUI: Graphical user interface
HGNC: HUGO gene nomenclature committee
HNSC: Head and neck squamous cell carcinoma
HTTP: Hyper text transfer protocol
HUGO: Human genome organisation
ICGC: International cancer genome consortium
KICH: Kidney chromophobe
KIRC: Kidney renal clear cell carcinoma
KIRC: Kidney renal clear cell carcinoma
KIRP: Kidney renal papillary cell carcinoma
LAML: Acute myeloid leukemia
LGG: Brain lower grade glioma
LGG: Lower grade glioma
LIHC: Liver hepatocellular carcinoma
LUAD: Lung adenocarcinoma
LUAD: Lung adenocarcinoma
LUSC: Lung squamous cell carcinoma
MESO: Mesothelioma
miRNA: microRNA
NCBI: National center for biotechnology information
OV: Ovarian serous cystadenocarcinoma
PAAD: Pancreatic adenocarcinoma
PCPG: Pheochromocytoma and paraganglioma
PRAD: Prostate adenocarcinoma
READ: Rectum adenocarcinoma
REST: REpresentational State transfer
RNA: Ribonucleic acid
SAM: Sequence alignment/map
SARC: Sarcoma
SKCM: Skin cutaneous melanoma
STAD: Stomach adenocarcinoma
TCGA: The cancer genome atlas
TGCT: Testicular germ cell tumors
THCA: Thyroid carcinoma
THYM: Thymoma
UCEC: Uterine corpus endometrial carcinoma
UCS: Uterine carcinosarcoma
UCSC: University of California at Santa Cruz
URL: Uniform resource locator
UUID: Universally unique identifier
UVM: Uveal melanoma
VCF: Variant call format
XML: eXtensible markup language
